# The closer the better: Hand proximity dynamically affects letter recognition accuracy

**DOI:** 10.3758/s13414-012-0339-3

**Published:** 2012-07-10

**Authors:** Jos J. Adam, Thamar J. H. Bovend’Eerdt, Fleur E. P. van Dooren, Martin H. Fischer, Jay Pratt

**Affiliations:** 1 Maastricht University Medical Centre, Maastricht University, P.O. Box 616, 6200 MD Maastricht, The Netherlands; 2 University of Potsdam, Potsdam, Germany; 3 University of Toronto, Toronto, Ontario Canada

**Keywords:** Perception and action

## Abstract

A growing literature has suggested that processing of visual information presented near the hands is facilitated. In this study, we investigated whether the near-hands superiority effect also occurs with the hands moving. In two experiments, participants performed a cyclical bimanual movement task requiring concurrent visual identification of briefly presented letters. For both the static and dynamic hand conditions, the results showed improved letter recognition performance with the hands closer to the stimuli. The finding that the encoding advantage for near-hand stimuli also occurred with the hands moving suggests that the effect is regulated in real time, in accordance with the concept of a bimodal neural system that dynamically updates hand position in external space.

In recent years, several studies have documented that positioning one or two hands near the location of stimuli can alter various aspects of visual cognition. Reed, Grubb, and Steele (2006) found that visual targets were detected faster when they appeared near a single hand placed on the side of the computer monitor. Subsequently, Abrams, Davoli, Du, Knapp, and Paull (2008) reported a variety of attentional effects when the (two) hands were proximal, as opposed to distal, to the display: steeper search slopes, greater inhibition of return, and greater attentional blink. In a four-choice reaction time task, Adam et al. (2008) found faster reaction times when the index and middle fingers of both hands were positioned near the stimulus locations on the computer monitor than when they were positioned on the keyboard. More recently, Tseng and Bridgeman (2011) reported superior performance in a change detection paradigm when the changed object was near the hands. Together, these and other studies (e.g., Cosman & Vecera, 2010; Dufour & Touzalin, 2008; Gozli, West, & Pratt, 2011; Reed, Betz, Garza, & Roberts, 2010) comprise a strong body of evidence indicating an enhancement of vision for stimuli presented near, as opposed to away from, the hands.

It has been proposed that these findings reflect a detailed *evaluation* of objects near the hand, either for potential manipulation or for a defensive response (e.g., Abrams et al., 2008). As was pointed out by Bridgeman and Tseng (2011), this interpretation is plausible, but additional scrutiny seems warranted, as most of the evidence is based on reaction time measures (e.g., Abrams et al., 2008; Adam et al., 2008; Reed, Grubb, & Steele, 2006) or has required the critical involvement of visual short-term memory (Tseng & Bridgeman, 2011). Reaction time measures in this context have two potential disadvantages. First, they allow the possibility that at least part of the facilitation is due to response-related factors—for instance, a lower criterion for responding to stimuli, instead of faster perception of them (Dufour & Touzalin, 2008). Second, tasks with reaction time measures sometimes call for a lateralized response (e.g., for pressing the key on the right side), which may introduce stimulus–response compatibility as a confounding factor (Lloyd, Azañón, & Poliakoff, 2010). In the present study, we sought to provide more direct evidence for an “enhanced visual analysis” account of the facilitative near-hands effect by examining recognition performance in a visual letter identification task, using response accuracy as the outcome measure.

A second important goal of this study was to determine whether the near-hands advantage also occurs with the hands moving (away from and toward the stimulus display). All previous studies on this topic have used static hands, but in daily life, perceptual–motor behavior often requires the flexible adaptation of body posture and of hand position to meet the demands of a changing environment. Furthermore, investigating the near-hands effect in a dynamic situation would allow the opportunity to test the bimodal-neuron hypothesis raised by Reed et al. (2006). According to this hypothesis, the near-hands superiority effect has its neural basis in bimodal neurons, which increase the neural saliency and representational power of objects located near the hand(s). These multisensory neurons react to both visual and tactile signals in peripersonal space (e.g., Graziano & Gross, 1994) and take limb position into account via proprioceptive signals (e.g., Brozzoli, Cardinali, Pavani, & Farnè, 2010). In particular, bimodal neurons in the posterior parietal cortex whose receptive fields *move with the hands* were considered as potential modulators of the near-hands effect (Reed et al., 2006). An important feature of bimodal neurons is that their response is spatially graded. As the visual stimulus appears increasingly farther away from the hand, the neurons fire progressively less. Hence, according to the bimodal-neuron hypothesis, hand proximity effects should be graded with the distance between the hands and the visual stimuli, as has been observed in at least one study (Reed et al., 2006).

In the present study, we evaluated the bimodal-neuron hypothesis of the near-hands phenomenon by examining hand position effects on letter identification performance in static and dynamic conditions of the hands. In the static condition, participants adopted a static body posture by keeping their two hands still in one of three positions (near, intermediate, or far away from the stimulus display). In the dynamic condition, participants continuously changed their posture by moving their hands toward and away from the stimulus display in a self-paced, cyclical manner, while concurrently performing the visual letter identification task. According to the bimodal-neuron hypothesis, graded hand proximity effects should be observed in the static as well as the dynamic hand conditions. Note that the dynamic hand condition would be particularly informative in this context, because a demonstration that the near-hands effect is modulated by *real-time* motor action would indicate the involvement of a dynamic multisensory-coding system for near-hand space, as postulated by the bimodal-neuron hypothesis. Finally, in both of our experiments, we prevented viewing of the hands by occlusion in order to demonstrate the influence of sensorimotor, as opposed to visual, information in the near-hands effect. In a similar vein, in the moving-hands condition, the to-be-identified letter displays were presented randomly throughout the movement trajectory in order to increase the reliance on real-time sensorimotor information regarding hand position.

## Experiment 1: Three-letter displays

In Experiment 1, we investigated the effect of hand position on the recognition of briefly presented three-letter arrays in static and dynamic hand conditions. That is, participants performed the recognition task with their hands either stationary or continuously moving toward and away from the stimulus display. We selected three hand positions at the moment of stimulus presentation: hands nearby, intermediate, or far away from the stimulus display. If, as the bimodal-neuron hypothesis assumes, static and dynamic hand location influences object recognition accuracy in real time and in a graded way, then near hands should produce more accurate letter identification than would far hands in both the static and dynamic conditions, with performance for the intermediate hand position lying in between.

### Method

#### Participants

A group of 20 students from Maastricht University (8 women, 12 men), with a mean age of 22.4 years (range: 20–29 years) participated. They were paid a small amount of money, had normal or corrected-to-normal vision, and were naïve as to the purpose of the experiment. The experiment was approved by the Ethics Committee of the Faculty of Psychology and Neuroscience at Maastricht University, and all participants gave written informed consent in accordance with the Declaration of Helsinki.

#### Apparatus, stimuli, and procedure

**Fig. 1:**
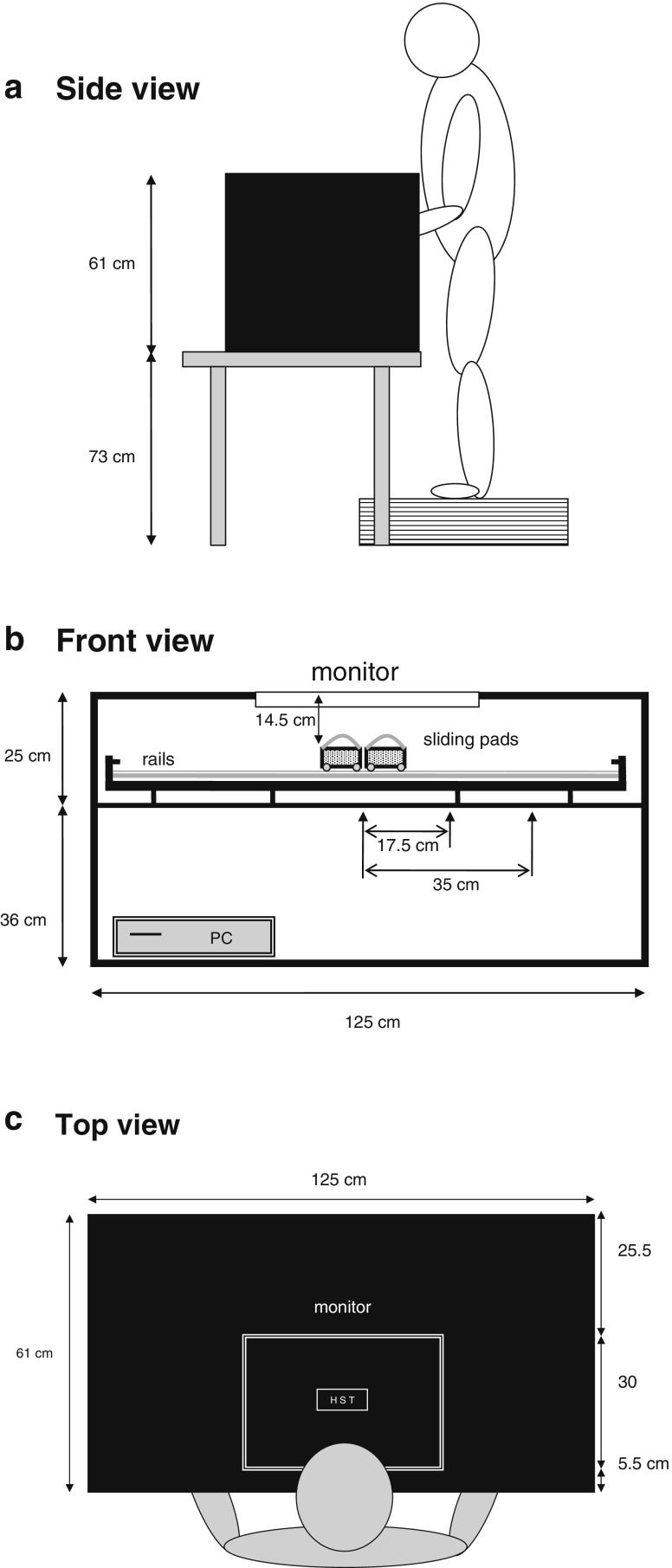
**A** Side view of the experimental setup. **B** Front view of the experimental setup. **C** Top view of the experimental setup. The computer monitor was placed in a horizontal position above the moving-hands apparatus, which consisted of two moveable keypads (one for each hand) that could slide over a rail. The position of the keypads in the figure reflects the near hands condition (center–keypad distance 0 cm). The intermediate hands condition (center–keypad distance 17.5 cm) and far hands condition (center–keypad distance 35 cm) are indicated by arrows. Not drawn to scale

Participants stood on a height-adjustable platform in front of the test apparatus, which contained a horizontally integrated 22-in. LCD computer screen (see Fig. 1a). They rested their hands on two moveable keypads mounted on a rail underneath the computer screen (see Fig. 1b). When viewing the monitor from above, the hands were invisible (see Fig. 1c). The hands were comfortably strapped to the pads with Velcro bands.

The stimuli consisted of an array of three different white letters, appearing in a white rectangular frame (58 × 30 mm) on a black background in the middle of the monitor. The individual letters measured approximately 6 × 6 mm and were separated by 6 mm. Viewing distance was about 35–40 cm. The stimuli were presented for one of five different short stimulus durations (27, 40, 53, 66, and 80 ms) and were then replaced with a masking stimulus (a row of five stars) for 200 ms. The participants were instructed to identify and report verbally as many letters as possible. The instructions emphasized that the identity of the letters was important (not the location) and that guessing was allowed if one was uncertain. The letters for each stimulus array were selected from all consonants (except the letters “Y” and “M”), making 19 different letters. The participant’s responses were written down by the experimenter and recorded by a voice recorder for cross-checking.

Half of the participants performed in the dynamic condition (moving hands) on the first day and the static condition (hands still) on the second day. The other half received the reverse order. In each session, the participants received three series of 19 trials (one for each hand position) for each of the five stimulus durations. In each series of 19 trials, each letter appeared three times in random order, with the constraint that each trial always contained three different letters. The order of the stimulus durations was random.

In the static condition, the two keypads were immobilized (i.e., fixed to the bar) with a center-to-keypad distance of 0 cm (near condition), 17.5 cm (intermediate condition), or 35 cm (far condition). Note that in the near condition, the hands were positioned directly underneath the center of the computer monitor where the stimulus array would appear. In the intermediate and far hand position conditions, the hands were progressively farther away from the stimulus location.

In the dynamic (moving-hands) condition, participants continuously moved their hands inward and outward at a comfortable frequency in a self-paced, rhythmic manner (Exp. 1: mean frequency  =  0.45 Hz, *SD* = 0.10; mean amplitude = 34.7 cm, *SD* = 0.6 cm; Exp. 2: mean frequency = 0.46 Hz, *SD* = 0.06; mean amplitude = 34.7 cm, *SD* = 0.3 cm). The two keypads were mechanically constrained such that the two hands always moved in opposite directions. Thus, participants performed mirror-symmetrical hand movements, which are easy to control because they require the simultaneous activation of the same muscle groups on both limbs (Swinnen, 2002). The time interval between successive stimulus arrays varied randomly between 3 and 4 s. Hence, in the dynamic condition, the location of the (moving) hands at the moment of stimulus presentation was variable, to be classified offline (i.e., after the experiment) as belonging to a near (center-to-keypad distance: 0–9 cm), intermediate (center-to-keypad distance: 9–26 cm), or far (center-to-keypad distance: 26–44 cm) hand position category, so that each category contained about one third of the trials (i.e., 33.6 %, 34.8 %, and 31.5 %, respectively). The time uncertainty with respect to stimulus presentation relative to hand position in the dynamic hand condition was implemented to maximize the need for online registration of hand position. The response pads were interfaced with the computer, which recorded the position of the hands online to the nearest 0.1 mm.

### Results and discussion

Mean percentages of correctly identified letters were calculated for each participant as a function of hand condition (static or dynamic), hand position (near, intermediate, or far), stimulus duration (27, 40, 53, 66, or 80 ms), and letter position (left, middle, or right). A repeated measures analysis of variance (ANOVA) was conducted on the mean percentages of correctly identified letters, with hand condition, hand position, stimulus duration, and letter position as within-subjects variables. Whenever appropriate, the tests were adjusted for heterogeneity of the variances and covariances using the Greenhouse–Geisser-corrected significance values. As expected, longer stimulus durations improved recognition performance [*F*(4, 76) = 51.87, *p* < .001, *η* ^2^ = .73; *M*s = 79.7 %, 85.4 %, 86.9 %, 89.8 %, and 90.5 %, respectively, for increasingly longer durations]. There was also a main effect of letter position, indicating that left letters were recognized better than middle and right letters [*F*(2, 38) = 10.17, *p* < .001, *η* ^2^ = .35; *M*s = 92.0 %, 84.0 %, and 83.4 % for left, middle, and right letters, respectively]. The advantage of the first letter position decreased with longer stimulus durations [see Table 1; *F*(8, 152) = 3.43, *p* < .01, *η* ^2^ = .15].

**Table 1:** Percentages of letters reported correctly as a function of stimulus duration and letter position in Experiment 1

Letter Position	Stimulus Duration (ms)				
	27	40	53	66	80
Left	87.4	91.9	92.7	94.8	95.5
Middle	74.9	82.6	83.1	87.6	88.9
Right	76.4	82.6	84.7	87.0	87.7

**Fig. 2:**
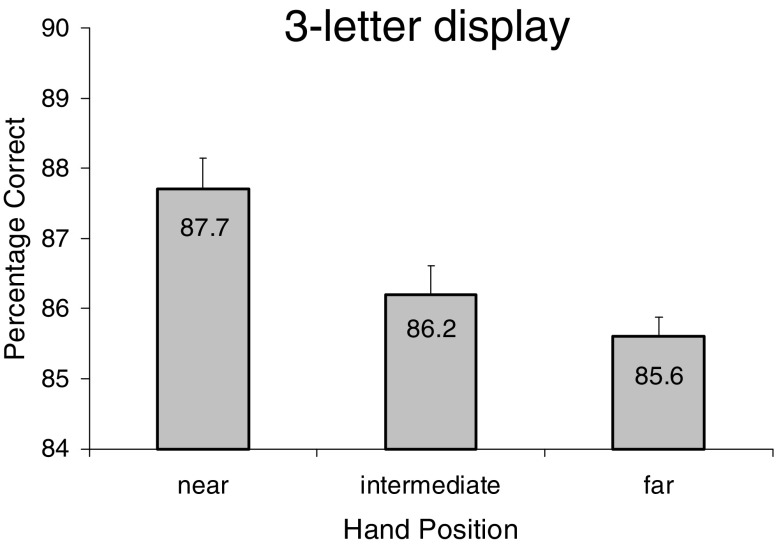
Mean percentages of letters reported correctly from the three-letter arrays as a function of hand position in Experiment 1. Error bars are within-subjects standard errors of the means

Most importantly, we found a significant main effect of hand position [*F*(2, 38) = 5.33, *p* < .01, *η* ^2^  =  .22]. Planned comparisons indicated superior letter recognition with the hands near the stimulus display, as compared with the hands intermediate (*p* < .05, one-tailed) and far away (*p* < .001, one-tailed) from the display, as can be seen in Fig. 2. No other effects reached significance. There was no main effect of hand condition [*F*(1, 19) = 2.28, *p* > .1, *η* ^2^ = .11], nor did hand condition interact with any other variable (*F*s < 2.42, *p*s > .05).

In sum, the key outcome of Experiment 1 was a graded improvement in the recognition of three-letter strings when the (invisible) hands were closer to the target display, regardless of whether the hands were moving or not. This finding extends previous demonstrations of enhanced visual processing of stimuli near the hands to letter identification accuracy. Importantly, the finding that the near-hands effect was present in a graded way and also when the hands were moving supports a key role of the bimodal neuron system in producing the effect, as hypothesized by Reed et al. (2006).

Despite the finding of a significant hand position effect, its magnitude was small (an absolute improvement of 2.1 %). Perhaps, with an overall accuracy rate of 86.5%, the task might have been relatively easy. In the next experiment, we increased task difficulty in an attempt to boost the sensitivity of the identification task to further examine hand position and hand condition (static vs. dynamic) effects.

## Experiment 2: Six-letter displays

In Experiment 2, we increased the task difficulty by using stimulus arrays that contained six different letters, three red and three white, presented in random order. Participants were asked to report the identity of the white letters only and to ignore the red letters. As compared to the three-letter displays used in Experiment 1, the six-letter, two-color displays placed greater demands on (attentional) selectivity. As in Experiment 1, participants performed the letter identification task with their hands either moving or still.

### Method

#### Participants

A group of 20 new students from Maastricht University volunteered (10 women, 10 men; mean age = 23.3 years, range 19–28 years).

#### Stimuli

The stimuli consisted of one row of six different letters, three white and three red, appearing in a white rectangular frame (100 × 30 mm) on a black background in the middle of the monitor. Red and white letters were randomly intermingled. Individual letters were approximately 6 × 6 mm, and separated by 6 mm. The stimulus durations were 95, 110, 125, 140, and 155 ms. Following stimulus offset, a masking stimulus (a row of 11 stars) was displayed for 200 ms, and the time between successive stimulus arrays varied randomly between 4 and 5 s. Participants were instructed to report the identity of the three white letters.

#### Apparatus and procedure

The apparatus and procedure were the same as in Experiment 1.

### Results and discussion

**Fig. 3:**
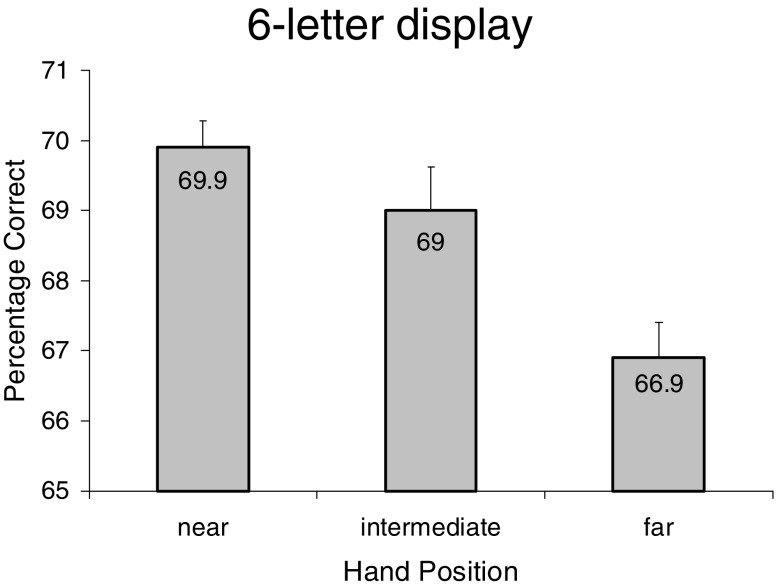
Mean percentages of (white) letters reported correctly from the six-letter arrays as a function of hand position in Experiment 2. Error bars are within-subjects standard errors of the means

As in Experiment 1, moving or not moving the hands was of no influence on recognition accuracy [*F*(1, 19) < 1]. Longer stimulus durations again improved performance [*F*(4, 76) = 52.73, *p* < .001, *η* ^2^ = .74; *M*s = 60.8 %, 66.2 %, 69.4 %, 72.0 %, and 74.5 %, respectively, for increasing durations]. The significant main effect of letter position indicated that the leftmost (white) letter was recognized best and the rightmost (white) letter was recognized worst [*F*(2, 38) = 52.51, *p* < .001, *η* ^2^ = .73; *M*s = 83.2 %, 66.0 %, and 56.6 % for the left, middle, and right letters, respectively; for all pairwise comparisons, *p* < .001], reflecting a serial, left-to-right scanning or read-out mechanism (e.g., Tydgat & Grainger, 2009). Furthermore, the advantage of the first letter position leveled off with longer stimulus durations [see Table 2; *F*(8, 152) = 2.37, *p* < .05, *η* ^2^ = .11]. Most critically, as in Experiment 1, there was a significant main effect of hand position [*F*(2, 38) = 5.70, *p* < .02, *η* ^2^ = .23]. Planned comparisons indicated better letter recognition for the near and intermediate hand positions than for the far hand position (*p*s < .001 and .05, one-tailed, respectively; see Fig. 3). No other effects reached significance.

**Table 2:** Percentages of letters reported correctly as a function of stimulus duration and letter position in Experiment 2

Letter Position	Stimulus Duration (ms)				
	95	110	125	140	155
Left	76.0	82.4	85.6	85.4	86.8
Middle	58.4	63.0	65.4	69.7	73.5
Right	48.1	53.4	57.1	60.9	63.2

The overall performance level in Experiment 2 was substantially lower than that in Experiment 1 [*M*s = 68.6 % vs. 86.5 %, respectively; *t*(38) = 5.55, *p* < .001], indicating that adding competing (distractor) letters to the stimulus display greatly increased task difficulty, possibly due to crowding (lateral inhibition; see, e.g., Bouma, 1970) and greater demands on selective spatial attention (e.g., Auclair & Siéroff, 2011). Furthermore, in Experiment 2, the near-hands advantage (relative to the far position) tended to be of greater *proportional* magnitude than that found with the three-letter displays in Experiment 1 [4.5 % vs. 2.4%, respectively; *t*(38) = –1.49, *p* = .07, one-tailed]. Finally, as in Experiment 1, the superior letter recognition performance in the near as compared to the far hand position was found in both static and dynamic hand conditions, which is consistent with the bimodal-neuron hypothesis.

## General discussion

This study investigated the effects of hand position (near, intermediate, or far) and hand condition (static vs. moving) on letter recognition performance by asking participants to identify three briefly presented, masked target letters, presented either alone (Exp. 1) or intermixed with three nontarget distractor letters in a different color (Exp. 2). The data of both experiments indicate that the accuracy of letter identification improves with the hands closer to the stimulus display. This novel finding extends previous reports of near-hands superiority effects found in other paradigms, such as visual detection, visual search, inhibition of return, attentional blink, figure–ground assignment, and change detection. Our finding is important because most of these earlier reports relied on reaction time measures, making it difficult to rule out response-speed-related factors. Hence, the present demonstration of improved letter identification accuracy near the hands reflects a facilitative effect on perceptual encoding processes, which fits with the suggestion that the enhancement of vision for near-hand objects reflects a mechanism that facilitates the detailed evaluation of objects for potential interaction (Abrams et al., 2008).

Two other key findings emerged from this study: The perceptual facilitation also occurred with the hands moving, and, moreover, in a graded way. Both of these findings are consistent with the dynamic and graded response features of the bimodal neuron system, which serves to provide online, multisensory coding of peripersonal action space (Graziano & Gross, 1994). These visuotactile integration processes mediate the orienting of spatial attention and generate a flexible, prioritized representation of space around the body. Moreover, they take into account changes of (limb) posture via proprioceptive signals and can be dynamically modulated by sensorimotor experiences, such as those associated with tool use (for a review, see Macaluso & Maravita, 2010).

It is worth noting, however, that the facilitative effects, although significant, were relatively small in magnitude. For one thing, in our study, the hands were positioned underneath the monitor and not at the side(s) of the monitor, as had been the case in most previous studies. Furthermore, a greater facilitative effect has been reported with the hands visible (e.g., Lloyd, Shore, Spence, & Calvert, 2003; Reed et al., 2006), suggesting further modulation of the facilitatory mechanism through visual hand position coding. Finally, in the present study the palms of the hands were facing downward—that is, away from the stimulus display. This factor, too, may have reduced the facilitative impact of the nearby hand condition, because greater facilitation has been reported for targets appearing near the palm of the hand, as compared to near the back of the hand (Reed et al., 2010).

Finally, in both experiments, we observed similar letter identification performance in static and moving hand conditions. This outcome indicates that moving the hands did not interfere with concurrently performing the letter identification task, suggesting independent, or at least interference-free, control (for a similar demonstration with keypress responses performed concurrently with moving the hands, see Adam & Moresi, 2007). This is in line with the notion of two different visual systems in the brain, variably called the “cognitive” and “sensorimotor” systems (e.g., Bridgeman, Peery, & Anand, 1997) or the “what” and “how” systems (e.g., Milner & Goodale, 1995), which are mediated by distinct neural pathways (a ventral and a dorsal pathway, respectively). Nevertheless, the present demonstration of perceptual facilitation due to real-time motor action suggests an inextricable linkage between perception and action at some elementary level. This function may be served by the bimodal neuron system, which integrates visual, tactile, and proprioceptive information into a dynamic and flexible representation of space around the body.
